# Regulation of Chitinase in *Spodoptera exigua* (Hübner) (Lepidoptera: Noctuidae) During Infection by *Heliothis virescens* ascovirus 3h (HvAV-3h)

**DOI:** 10.3389/fphys.2020.00166

**Published:** 2020-03-10

**Authors:** Lei He, Yi-Yi Ou-Yang, Ni Li, Ying Chen, Shuang-Qing Liu, Guo-Hua Huang

**Affiliations:** ^1^Hunan Provincial Key Laboratory for Biology and Control of Plant Diseases and Insect Pests, Hunan Agricultural University, Changsha, China; ^2^College of Plant Protection, Hunan Agricultural University, Changsha, China

**Keywords:** heliothis virescens ascovirus 3h, transcriptional pattern, chitinases, chitin-binding domain, *Spodoptera exigua*

## Abstract

Insect chitinases play essential roles in the molting and metamorphosis of insects. The virus Heliothis virescens ascovirus 3h (HvAV-3h) can prolong the total duration of the larval stage in its host larvae. In this study, the molecular character and function of chitinase and chitin-binding domain (CBD) were analyzed in larvae of *Spodoptera exigua* (Hübner) (Lepidoptera: Noctuidae). In detecting the chitinase activity of mock-infected and HvAV-3h-infected larval whole bodies and four different larval tissues, the results showed that larval chitinase activity was significantly decreased at 48 h post infection (hpi) and that the chitinase activity of HvAV-3h-infected larval fat body and cuticle was notably decreased at 144 and 168 hpi. The transcription level of *S. exigua chitinase* 7 (*SeCHIT7*) was down-regulated at the 6, 9, 12, 48, 72, and 96 hpi sample times, the *S. exigua chitinase* 11 (*SeCHIT11*) was down-regulated at 3–96 hpi, while both *S. exigua chitinases* (*SeCHIT*s) were up-regulated at 120–168 hpi. Further tissue-specific detection of *SeCHIT7* and *SeCHIT11* transcription showed that *SeCHIT7* was down-regulated at 144 and 168 hpi in the fat body and cuticle. *SeCHIT11* was down-regulated at 168 hpi in the fat body, midgut, and cuticle. Additionally, the transcription and expression of *S. exigua* chitin-binding domain (*SeCBD*) could not be detected in HvAV-3h-infected larvae. The *in vitro* analyses of SeCHIT7N, SeCHIT11, and SeCBD showed that SeCHIT7N and SeCHIT11 were typical chitinases. Conversely, no chitinase activity was detected with SeCBD. SeCBD, however, could significantly increase the activity of SeCHIT7N and SeCHIT11. In conclusion, HvAV-3h not only interfered with the transcription and expression of *SeCHIT*s but also affected the normal transcription and expression of *SeCBD* and, in doing so, influenced the host larval chitinase activity. These results will aid in providing a foundation for further studies on the pathogenesis of HvAV-3h.

## Introduction

Insect chitinases are a class of hydrolases for degrading chitin – the main component of insect cuticle and peritrophic membrane (PM) (Hegedus et al., 2009). To overcome the rigid constraints of chitinous exoskeletons, insects undergo periodic molting to accommodate growth (Merzendorfer and Zimoch, 2003; Zhu et al., 2008b). In molting and metamorphosis, chitinases exhibit periodic changes while playing indispensable roles (Fukamizo, 2000; Merzendorfer and Zimoch, 2003; Wu et al., 2013). Since the first insect chitinase gene was identified from *Manduca sexta*, many additional chitinase genes have been discovered (Kramer et al., 1993) and subsequently classified into eight groups. Among these, chitinase 7 belongs to the extensively studied group III chitinases. For example, *Sogatella furcifera* chitinase 7 (*SfCHIT7*) was highly transcribed in the day 3 fifth-instar nymphs and newly emerged adults of its host. *Drosophila melanogaster* chitinase 7 (*DmCHIT7*) showed a broad transcriptional level in pupae, similar to *Anopheles gambiae* chitinase 7 (*AgCHIT7*), which also showed the highest transcription level during the pupal stage; *Tribolium castaneum* chitinase 7 (*TcCHIT7*) was mainly transcribed in the last instar larvae, pupae, and adults (Zhu et al., 2008a; Zhang et al., 2011; Pesch et al., 2015; Chen et al., 2017). Our previous research showed that *Spodoptera exigua* chitinase 7 (*SeCHIT7*) and *S. exigua* chitinase 11 (*SeCHIT11*) were strongly transcribed during pupation and eclosion (He et al., 2019), which suggests that chitinase plays an indispensable role in these processes. In addition, chitin-binding domain (CBD) is important for forming, maintaining, and regulating the functions of chitin structures (Tetreau et al., 2015). CBD is required for chitinase to bind specifically to insoluble chitin and to hydrolyze it efficiently (Watanabe et al., 1990, 1994). On the one hand, CBD may enhance chitinase activity by concentrating the chitinase on a chitinous surface (Gilkes et al., 1988; Tomme et al., 1988), while on the other; CBD may disrupt non-covalent interactions including hydrogen bonds between adjacent glucose units (Din et al., 1991). CBD can effectively enable chitinase to degrade insoluble polysaccharides into soluble oligosaccharides during the molting process (Arakane et al., 2003).

It has been reported that pathogens can cause changes in the transcriptional pattern or activity of chitinases during the infection process. One example of this is where infection with *Beauveria bassiana* leads to a great variation in chitinase activity in *Hypothenemus hampei* (Acuña-Payano et al., 2017). Another instance was reported by Zhou et al. (2017) in black tiger shrimp (*Penaeus monodon*), where *Streptococcus agalactiae* and *Vibrio harveyi* significantly affected the transcriptional level of *Penaeus monodon* chitinase 4 (*PmCHIT4*) in different tissues and developmental stages. Because changes in chitinases following infection by pathogens have been shown to influence the growth and development of hosts, analyzing the transcriptional profiles and activity of chitinase in a host after being infected by the pathogens is a critical step in studying the effect of pathogens on the growth and development of their hosts.

Ascoviruses, members of the virus family *Ascoviridae*, are known to primarily infect lepidopteran larvae of the family Noctuidae (Chen et al., 2017; Yu et al., 2019). Their genome consists of a circular, double-stranded DNA molecule ranging in size from 100 to 200 kbp (Federici et al., 1990; Bigot et al., 1997; Cheng et al., 1999; Chen et al., 2018). Although they likely occur worldwide, they have been reported most frequently from field populations in the Americas, Australia, Indonesia, China, and Japan. To date, the genomes of nine ascoviral isolates have been sequenced and reported: Spodoptera frugiperda ascovirus 1a (SfAV-1a) (Bideshi et al., 2006), Trichoplusia ni ascovirus 6a (TnAV-6a) (Wang et al., 2006), Heliothis virescens ascovirus 3e (HvAV-3e) (Asgari et al., 2007), Heliothis virescens ascovirus 3g (HvAV-3g) (Huang et al., 2012b), Heliothis virescens ascovirus 3f (HvAV-3f) (Wei et al., 2014), Heliothis virescens ascovirus 3h (HvAV-3h) (Huang et al., 2017), Heliothis virescens ascovirus 3i (HvAV-3i) (Chen et al., 2018), Heliothis virescens ascovirus 3j (HvAV-3j) (Arai et al., 2018), and Trichoplusia ni ascovirus 6b (TnAV-6b) (Liu et al., 2018). According to the above genome reports, these ascoviruses did not encode chitinase genes in the genome. In China, the virus HvAV-3h, primarily infecting *Helicoverpa armigera*, *S. exigua*, and *S. litura* larvae, has been isolated (Huang et al., 2012a). HvAV-3h can prolong the lifespan and inhibit pupation in infected host larvae (Li et al., 2013; Hu et al., 2016). According to the reported genome, however, HvAV-3h did not encode the gene for chitinase (Huang et al., 2017). Therefore, HvAV-3h infection may be closely related to the transcriptional pattern or activity of chitinase encoded by its host.

In order to investigate the effect of HvAV-3h on chitinase, tests to detect the transcription and expression of *SeCHIT7*, *SeCHIT11*, and *SeCBD* were conducted on mock-infected and infected larvae. Additionally, the chitinase activity of SeCHIT7N, SeCHIT11, and SeCBD and the effect of SeCBD on SeCHIT7N and SeCHIT11 were analyzed *in vitro*. The results provided data with which to further understand the regulation of HvAV-3h on chitinase in *S. exigua* larvae during virus infection.

## Materials and Methods

### Experimental Insects and Preparation of Samples

*Spodoptera exigua* larvae were cultured in an artificial climate incubator with controlled temperature, humidity, and photoperiod (27 ± 2°C, humidity 70 ± 10%, and 14:10/L:D) as previously described (Li et al., 2015; Yu et al., 2020a, b). Heliothis virescens ascovirus 3h (HvAV-3h) was taken from a stock maintained in the laboratory (Huang et al., 2012a; Li et al., 2013).

#### Inoculating HvAV-3h and Obtaining Samples

A sterile insect pin dipped into hemolymph drawn from an HvAV-3h-infected larva was used to pierce a proleg of newly molted third instar larvae. The infected larvae were reared separately on artificial diet dots. Mock-infected larvae were handled identically except that the pins were dipped into hemolymph drawn from healthy larvae. Treated larval bodies were collected at 0, 3, 6, 9, 12, 24, 48, 72, 96, 120, 144, and 168 h post infection (hpi). They were immediately quick-frozen using liquid nitrogen and stored at −80°C. At 48, 72, 96, 120, 144, and 168 hpi, the treated larvae were dissected on ice to obtain various tissues, including hemolymph, fat body, midgut, and cuticle. The collected samples were immediately frozen as above. Each treated sample contained at least six larvae, and each treatment was replicated three times.

### Total RNA Extraction and cDNA Synthesis

In order to establish the effect HvAV-3h has on the transcriptional patterns of *SeCHIT*s, whole-body samples and various tissues were used in RNA extraction. Total RNA of all samples was extracted using TRI Reagent^®^ RNA/DNA/protein Isolation Reagent (Invitrogen, Cincinnati, OH, United States) according to the manufacturer’s instructions. The cDNA of various samples was generated from their total RNA using GoScript^TM^ Reverse Transcription Mix, Oligo (dT) (Promega Corp., Madison, WI, United States) according to the manufacturer’s instructions.

The annealing temperature was 55°C. The 2-μg cDNA was used as a template to amplify the *SeCHIT7N* with the forward primer (5′ GGATCCATGTGGCCACCAAGATTG 3′, a *Bam*HI site contained) and the reverse primer (5′ AAGCTTTTGACGGGCTTCTTA 3′, a *Hin*dIII site contained) according to the open reading frame predicted from ORF Finder^1^. *SeCHIT11* was amplified with primers SeCHIT11-F (5′ GGATCCATGGCGTTCCGATCG 3′, a *Bam*HI site contained) and SeCHIT11-R (5′ AAGCTTCTATTCCTCGTCGGCG 3′, a *Hin*dIII site contained). *SeCBD* was amplified with primers SeCBD-F (5′ GAGCTCATGTTTTGGAGTGCAGTG 3′, a *Sac*I site contained) and SeCBD-R (5′ AAGCTTTTAGGGGTTAACCTCACC 3′, a *Hin*dIII site contained). The PCR products were purified using an EasyPure PCR Purification Kit (TransGen Biotech Co., Ltd., Beijing, China) and then cloned into pGEM-T Easy Vector (Promega Corporation). Five white clones were selected and sent to TsingKe Biological Technology, Co., Ltd., (Changsha, Hunan, China) to confirm the clone by sequencing.

### Preparation of Polyclonal Antiserum

The *SeCHIT7N*, *SeCHIT11*, and *SeCBD* CDS fragments were digested from the constructed SeCHIT7N-T and SeCHIT11-T vectors with *Bam*HI and *Hin*dIII and the SeCBD-T vectors with *Sac*I and *Hin*dIII. Then, *SeCHIT7N* was ligated with pET-28a (+) vector (Novegen, Madison, WI, United States) and *SeCHIT11* and *SeCBD* with pET-32a (+) vector (Novegen) and then digested with the same enzymes to generate the prokaryotic expression vectors pET-28a-SeCHIT7N, pET-32a-SeCHIT11, and pET-32a-SeCBD, respectively. The resulting pET-28a-SeCHIT7N, pET-32a-SeCHIT11, and pET-32a-SeCBD vectors were transformed into *Escherichia coli* BL21 (DE3) followed by inducing with 1 mM isopropyl β-D-thiogalactoside (IPTG) at 25°C for 22 h. The scaled inducing bacteria were collected and destroyed via ultrasound in Balance Buffer (150 mM NaCl, 50 mM Tris-Cl, pH8.0, and 10 mM imidazole). After centrifugation at 12,000 g for 15 min, the supernatant was used for His-Tag fused protein affinity purification with ProteinIso Ni-NTA Resin (TransGen Biotech Co., Ltd., Beijing, China) according to the manufacturer’s instructions. The protein samples were loaded and separated by 12% SDS-PAGE followed by staining with Coomassie Blue.

The purified protein in complete Freud’s adjuvant (Sigma Chem. Corp., MO, United States) was injected subcutaneously to immunize New Zealand white rabbits. After another two booster injections in incomplete Freund’s adjuvant at 2-week intervals, the rabbits were exsanguinated. The prepared polyclonal rabbit antisera against SeCHIT7N, SeCHIT11, and SeCBD were used for the subsequent immunoassays.

### Determination of Chitinase Activity *in vivo*

Colloidal chitin was derived following the method of Nitoda et al. (1999). One gram of commercial chitin (Shanghai Yuanye Biological Technology Co., Ltd., Shanghai, China, Cat. No. S11065) was ground in a mortar with acetone and concentrated hydrochloric acid into a paste, and the paste was then precipitated with pre-cooled 50% ethanol. The colloidal precipitation that was obtained from the homogenate by sedimentation (4000 rpm × 5 min) was rinsed repeatedly with ddH_2_O until neutral. Samples obtained from the insects were homogenized by adding 1 mL PBS (0.2 M phosphate buffer, pH 6.4) and centrifuged (4°C, 10000 rpm, 15 min). The supernatant was the enzyme solution extracted from the body of the test insect (Boller et al., 1983). The protein content was assayed using a Bicinchoninic Acid (BCA) Protein Quantification Kit (Zhuhai Joincare Biosciences Ltd., Zhuhai, Guangdong, China) according to the manufacturer’s instructions, with the enzyme concentration diluted to 1 μg/mL.

**FIGURE 1:**
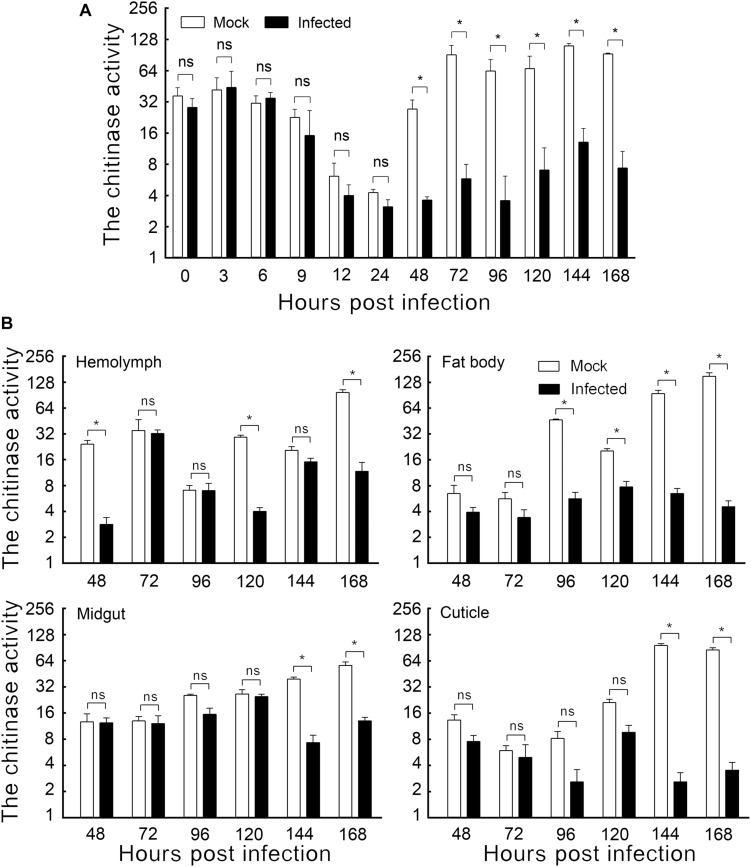
The effect of HvAV-3h on chitinase activity in *Spodopera exigua* larvae. **(A)** Chitinase activity in the whole body. **(B)** Chitinase activity in different tissues. Mock, mock-infected larvae; Infected, infected larvae. * indicates significant difference between mock-infected and infected (*p* < 0.05), ns indicates no difference between mock-infected and infected according to *t*-test.

The chitinase activity was determined by the method described by He et al. (2019). A 100 mL volume of the insect body extract enzyme solution was combined with 100 μL colloid chitin and 20 μL PBS and gently mixed by pipetting after each addition. The mixture was incubated at 40°C for 1 h, followed by immediately chilling in flowing water, and then centrifuged for 5 min (4°C, 10,000 rpm). A volume of 100 μL of the supernatant was added to 20 μL of 0.8 M potassium tetraborate and mixed thoroughly. The mixture was incubated in boiling water for 3 min and then immediately chilled in flowing water. The sample was then added to 600 μL of 4-dimethylaminobenzaldehyde (DMAB, 10 g DMAB dissolved in 100 mL glacial acetic acid containing 12.5% of 10 M hydrochloric acid). The sample was immediately mixed and incubated at 37°C for 20 min and then chilled in flowing water. A 200-μL volume of reaction product was used for determining the OD585. The enzyme activity was calculated, and the Log2Fold values are shown in Figure 1A.

### *In vitro* Chitinase Activity Assays

The chitinase activity of SeCHIT7N, SeCHIT11, and SeCBD were determined according to the above method with a slight modification: 100 μL insect body extract enzyme solution was replaced by 100 μL exogenous expression protein solution. Standard curves of SeCHIT7N, SeCHIT11, and SeCBD were prepared by the enzyme-linked immunosorbent assay (ELISA) method. Total protein was obtained by fragmentation of *E. coli* BL21 (DE3) containing SeCHIT7N, SeCHIT11, and SeCBD with glass beads (Shanghai Sangon Biotech Co., Ltd., Shanghai, China). Calibration of the SeCHIT7N, SeCHIT11, and SeCBD content in total protein was performed by using standard curves. To measure the effect SeCBD had on the activity of SeCHIT7N and SeCHIT11, the chitinase activity was determined after mixing SeCHIT7N or SeCHIT11 with SeCBD in 1:0, 1:1, 1:2, 1:5, 1:10, and 1:20 proportions.

### Transcription Analysis of *SeCHIT7N* and *SeCHIT11* in *S. exigua*

Quantitative real-time PCR (qRT-PCR) reactions were performed using 2 × SYBR green real-time PCR mix (TaKaRa) according to the manufacturer’s instructions. The 20-μL total reaction volume included 10 μL of 2 × SYBR green real-time PCR mix, 0.8 μL each of gene-specific primers, and 1 μL of cDNA templates, with ddH_2_O as necessary to the final volume. Glyceraldehyde-3-phosphate dehydrogenase (*gapdh*) was used as the reference gene (NCBI accession NO. JF728815.1). The reactions were conducted on a CFX96 Touch Real-Time PCR Detection System (Bio-Rad Laboratories, CA, United States). The annealing temperature was 55°C. We used the following primers: for *SeCHIT7*, forward, 5′-TTCACTCTGTCGGCTGGTA-3′, and reverse, 5′-CCTTCTTTGTCAACTTCGGTA-3′; for *SeCHIT11*, forward, 5′-AGGAGGCTGAAATCACGAA-3′, and reverse, 5′-AGCAGTCTAAGTTGAATGGC-3′; for *gapdh*, forward, 5′-GTCGTGTCATCCGATTTCATT-3′, and reverse, 5′-TAGCCAAACTCGTTGTCATACC-3′ (He et al., 2019). All samples were analyzed in triplicate and the relative target gene transcription levels calculated using the 2^–ΔΔ*Ct*^ method (Livak and Schmittgen, 2001).

Because of the characteristics of *SeCBD*, the transcription of *SeCBD* cannot be detected by qRT-PCR. Instead, the transcription of *SeCBD* was detected by semi-quantitative polymerase chain reaction with primers SeCBD-F (ATGTTTTGGAGTGCAGTG) and SeCBD-R (TTAGGGGTTAACCTCACC) in *S. exigua*, with *Segapdh* used as the internal loading control (Yu et al., 2018).

### Expression Analysis of SeCHIT7N, SeCHIT11, and SeCBD in *S. exigua*

Proteins were extracted from the chloroform-extracted organic phase of each RNA prep sample (including the HvAV-3h-infected larval whole bodies and dissected tissues mentioned above) according to the TRI reagent manufacturer’s instructions (Molecular Research Center, Inc., OH, United States). The extracted protein samples were separated by the 12% SDS-PAGE system and transferred to a nitrocellulose membrane. The prepared SeCHIT7N (1:3000), SeCHIT11 (1:3000), and SeCBD (1:2000) polyclonal antiserum, as well as the antiserum of HvAV-3h-encoded major capsid protein (MCP, 1:3000), were used as the primary antibody. The horseradish peroxidase (HRP)-conjugated goat anti-mouse lgG (1:5000) (Proteintech Group, Hubei, China) was used as the secondary antibody. The moth’s specific GAPDH antibody (1:4000) was used as a reference antibody in insect cellular analysis (Yu et al., 2018). The proteins were visualized with Clarity^TM^ Western ECL Substrate (Bio-Rad).

### Statistical Analysis

Statistical significance of differences between treatments was analyzed by *t*-test. The data were analyzed using SPSS 22.0 software (IBM Corporation, NY, United States) and denoted as mean ± SE (standard error). Statistical significance was established as *p* < 0.05.

## Results

### Effect of HvAV-3h Infection on Host Larval Chitinase Activity

The enzyme activities of mock-infected and HvAV-3h-infected larval bodies were assayed to demonstrate the changes in chitinase activity caused by HvAV-3h. Slight but non-significant differences were detected within the first 24 hpi. Later sampling periods, however, did show significant differences. Larvae infected with HvAV-3h for 48, 72, 96, 120, 144, and 168 h had chitinase activities that were 13.20, 6.37, 5.63, 10.49, 11.79, and 7.94% that of mock-infected larvae, respectively (Figure 1A). The results indicated that HvAV-3h had substantial effects on chitinase activity, especially at 48, 72, 96, 120, 144, and 168 hpi.

To investigate the effect of HvAV-3h infection on chitinase activity in various tissues of *S. exigua* larvae, the hemolymph, fat body, midgut, and cuticle tissues of mock-infected and HvAV-3h-infected larvae were dissected and the chitinase activity measured in each of the samples. The results indicated that, compared to the mock-infected samples, hemolymph infected with HvAV-3h had dramatically reduced chitinase activity at 48, 120, and 168 hpi (Figure 1B). In the fat body, the infected larval chitinase activity was dramatically lowered than in mock-infected larvae at 96, 120, 144, and 168 hpi (Figure 1B). Compared to mock-infected *S. exigua* larvae, chitinase activity was significantly decreased at 144 and 168 hpi in the midgut and cuticle, while no significant differences were observed between infected and mock-infected larvae at 24, 48, 72, 96, and 120 hpi (Figure 1B). Collectively, the infected larval chitinase activity was substantially lower than in the mock-infected larvae at 144 and 168 hpi (the key period of pupation). At 168 hpi, chitinase activities in the hemolymph, fat body, midgut, and cuticle tissues were reduced by 87.95%, 96.96%, 76.84%, and 95.86%, respectively. In addition to severely affecting the activity of chitinase in each of the four tissues, particularly the fat body and cuticle, these chitinase reductions are thought to be associated with the observed protracted periods of time spent in the last larval instars, as well as preventing successful pupation by affected larvae.

### Effects of HvAV-3h on Transcription and Expression of *SeCHIT*s

**FIGURE 2:**
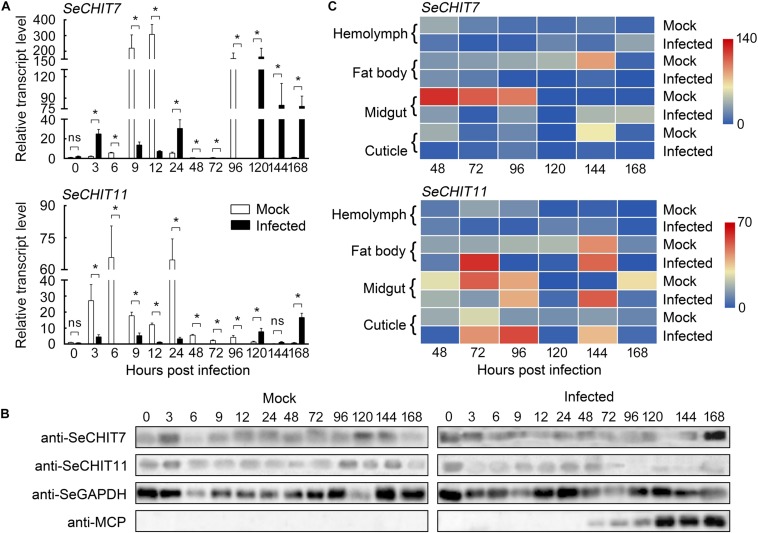
The effect of HvAV-3h on transcription and expression in *Spodopera exigua* larvae. **(A)** Relative transcriptional level of the *SeCHIT*s in larval whole bodies after HvAV-3h infection. **(B)** Expression of the SeCHITs in larval whole bodies. **(C)** Heat map of relative transcriptional level of the *SeCHIT*s in different tissues after HvAV-3h infection. Mock, mock-infected larvae; Infected, infected larvae. * indicates significant difference between mock-infected and infected (*p* < 0.05), ns indicates no difference in transcription level according to *t*-test.

To verify that HvAV-3h regulates the host’s growth and development by affecting chitinases encoded by the host, we first analyzed whether HvAV-3h produced changes in the transcriptional patterns of the chitinases in host larvae. The relative transcriptional levels of *SeCHIT7* and *SeCHIT11* were determined using cDNA prepared from whole-body samples from larvae subjected to the different treatments (Figure 2). Quantitative real-time PCR (qRT-PCR) results demonstrated that the transcriptional level of *SeCHIT7* was notably down-regulated at 6, 9, 12, 48, 72, and 96 hpi in infected larvae. At 96 hpi, there was a 99.94% reduction in HvAV-3h-infected larvae compared to mock-infected *S. exigua* larvae, while after 120 hpi, transcription of *SeCHIT7* induced by HvAV-3h-infected larvae was more than 1300-fold higher than levels in mock-infected larvae. *SeCHIT7* was also strongly up-regulated at 3, 24 144, and 168 hpi (Figure 2A). The transcriptional level of *SeCHIT11* was significantly decreased at 3, 6, 12, 24, 48, 72, and 96 hpi. At 6 hpi, it had decreased by 99.98% of the amount found in mock-infected larvae. After 168 hpi, however, it had increased by 25.15-fold compared with the mock-infected larvae (Figure 2A). The Western blot results showed that HvAV-3h infection severely affected the expression of the two chitinases (Figure 2B). These results indicated that HvAV-3h severely disrupted the normal transcriptional and expressional profiles of the two *SeCHIT*s in *S. exigua* larvae.

Our second sets of analyses were conducted to determine how HvAV-3h regulates the transcriptional profiles of *SeCHIT7* and *SeCHIT11* in the four selected tissues. Transcriptional levels of both chitinases were detected by qRT-PCR in the hemolymph, fat body, midgut, and cuticle of mock-infected and HvAV-3h-infected larvae at different hpi. The heat map of the genes’ transcriptional levels is shown in Figure 2C. The results show that the transcription of *SeCHIT7* was suppressed at 48, 72, and 96 hpi, while *SeCHIT11* was promoted at 120, 144, and 168 hpi in the hemolymph. The transcriptional level of *SeCHIT7* in infected larval hemolymph (Mean = 0.68 ± 0.12) was ca. 22.52% that of mock-infected larval hemolymph (Mean = 3.02 ± 0.34) at 48 hpi and was up-regulated by 8.26-fold at 168 hpi. The transcription of *SeCHIT11* was down-regulated by 86.84% at 72 hpi and up-regulated 10.13-fold at 120 hpi compared with the mock-infected larvae (Figure 2C). When infected with HvAV-3h, the transcription of *SeCHIT7* was significantly suppressed at 96, 120, and 144 hpi in the fat body, especially at 144 hpi, when it was down-regulated by 98.74% compared with the mock-infected insects (Mean = 0.39 ± 0.16) (Figure 2C). In the fat body, the transcriptional level of *SeCHIT11* was increased by 30.80-fold at 72 hpi compared with mock-infected larvae (Mean = 2.10 ± 0.37) (Figure 2C). After infection with HvAV-3h for 96 and 120 h, the relative transcript levels of *SeCHIT11* in the larval fat body were reduced to 1.45% and 4.35% that of mock-infected specimens (Figure 2C). After being infected with HvAV-3h for 48 and 72 h, the transcriptional level of *SeCHIT7* in the larval midgut was 98.49% and 99.88% lower than it was in mock-infected larvae. However, *SeCHIT7* was increased by 65.17-fold at 144 hpi, 56.13-fold at 168 hpi compared with the mock-infected larvae (Figure 2C). The transcription of *SeCHIT11* was suppressed by 95.95% at 72 hpi after infection by HvAV-3h, and the transcriptional level of *SeCHIT11* was increased by 700-fold at 144 hpi compared with the mock-infected larvae (Mean = 0.05 ± 0.03) (Figure 2C). The transcriptional level of *SeCHIT7* in the cuticle declined 97.41% at 144 hpi compared with mock-infected insects (Mean = 0.28 ± 0.06) (Figure 2C). The transcriptional level of *SeCHIT11* in HvAV-3h-infected larvae was only 7.08% that of mock-infected larvae at 48 hpi and increased to more than 34-fold that in mock-infected larvae at 96 hpi (Figure 2C). These results suggested that the transcriptional profiles of the two *SeCHIT*s are regulated by HvAV-3h in these four tissues, especially *SeCHIT7* in the fat body and cuticle.

### Effects of HvAV-3h on Transcription and Expression of *SeCBD*

**FIGURE 3:**
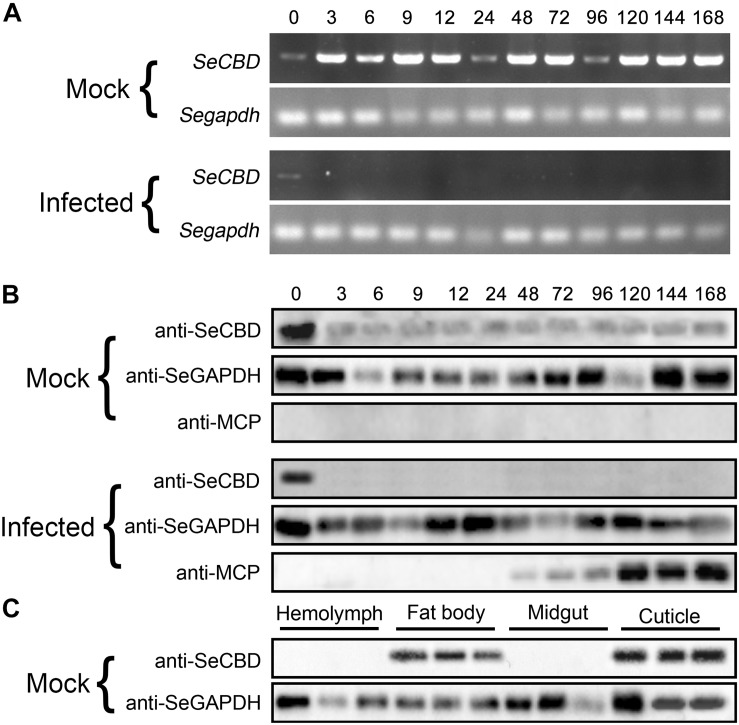
Comparison of *SeCBD* transcription and expression level in HvAV-3h-infected larvae and mock-infected larvae of *Spodopera exigua*. **(A)** *SeCBD* transcription level detection in HvAV-3h-infected larvae and healthy larvae. **(B)** SeCBD expression level detection in HvAV-3h-infected larvae and mock-infected larvae. **(C)** SeCBD expression level detection in healthy larval tissues. Mock, mock-infected larvae; Infected, infected larvae. Error bars represent standard deviation.

To identify the effects of HvAV-3h on transcription and expression of *SeCBD*, we determined the transcription and expression of *SeCBD* in mock-infected and infected whole bodies and the expression of *SeCBD* in each of the four mock-infected larval tissues. Because the transcriptional change of *SeCBD* could not be detected by qRT-PCR in infected larvae, semi-quantitative polymerase chain reaction was used instead in both mock-infected and infected larvae. Preliminary results had shown that normal transcription of *SeCBD* was detectable during the development of mock-infected larvae but not in HvAV-3h-infected larvae except at 0 hpi (Figure 3A). Similar results were obtained using Western blot (Figure 3B). The expression of SeCBD in mock-infected larval tissues was detected, showing that SeCBD was mainly expressed in cuticle and fat body but not in hemolymph and midgut (Figure 3C), indicating that HvAV-3h had strong effects on transcription and expression of *SeCBD*.

### Effects of SeCBD on Chitinase Activity of SeCHIT7N and SeCHIT11 *in vitro*

**FIGURE 4:**
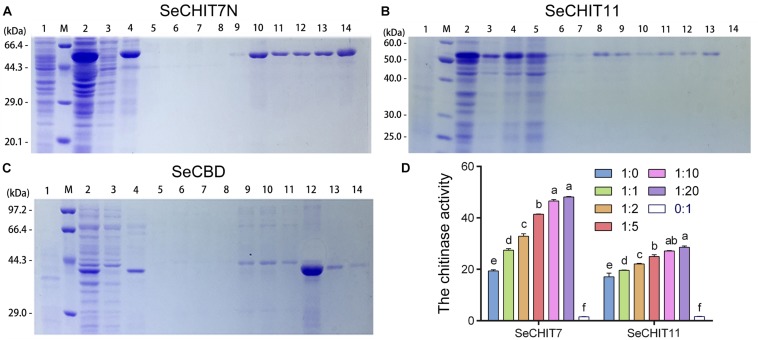
Detection interaction of SeCHITs and SeCBD by eukaryotic expression. **(A)** Expression and purification of SeCHIT7N. **(B)** Expression and purification of SeCHIT11. **(C)** Expression and purification of SeCBD. **(D)** Chitinase activity detection of SeCHIT7N, SeCHIT11, SeCBD, and SeCHITs + SeCBD. Error bars prepresent standard deviation.

SeCHIT7N was the N-terminal of SeCHIT7 and contained 511 amino-acid residues, with a molecular weight of approximately 60.65 kDa. With inducing by IPTG, approximately 62 kDa of fused SeCHIT7N was detected (Figure 4A, lane 2). After ultrasonication, almost all fused SeCHIT7N proteins appeared in the inclusion body-loaded lane (Figure 4A, lane 4). The inclusion body protein was dissolved with 8M urea. During the affinity purification, SeCHIT7N in the supernatant was eluted out from buffer C (Figure 4A, lane 9, 10), buffer D (Figure 4A, lanes 11–13), and buffer E (Figure 4A, lane 14). SeCHIT11 contained 299 amino-acid residues with a molecular weight of about 32.89 kDa. With inducing by IPTG, because the pET-32a (+) vector expressed a ∼20 kDa fusion protein, approximately 54 kDa of fused SeCHIT11 was detected (Figure 4B, lane 2). After ultrasonication, the supernatant contained a small amount of soluble protein (Figure 4B lane 3), and almost all fused SeCHIT11 proteins appeared in the inclusion body-loaded lane (Figure 4B, lane 4). The inclusion body protein was dissolved with 8M urea. During the affinity purification, SeCHIT11 in the supernatant was eluted out from buffer C (Figure 4B, lanes 8–10) and buffer D (Figure 4B, lane 11–13). With inducing by IPTG, approximately 43 kDa fusion protein fused SeCBD was detectable with the elution of buffer E (Figure 4C, lanes 12–14), and a high purity of SeCBD was obtained in lane 12. Finally, purified SeCHIT7N, SeCHIT11, and SeCBD were obtained for the subsequent studies.

After ultrasonication, almost all expressed proteins become inactive inclusion body protein. However, the total protein obtained by fragmentation of *E. coli* BL21 (DE3) containing SeCHIT7N, SeCHIT11, and SeCBD with glass beads contained protein with active enzymes. The chitinase activity of SeCHIT7N, SeCHIT11, and SeCBD was detected, and negative control was obtained by fragmentation of *E. coli* BL21 (DE3) solution without exogenous protein.

In order to illustrate the properties of SeCHIT7N, SeCHIT11, and SeCBD, their chitinase activities were determined first. The result showed that no chitinase activity was detected in SeCBD, while SeCHIT7N and SeCHIT11 exhibited high chitinase activity (Figure 4D). The presence of SeCBD significantly increased the activity of SeCHIT7N and SeCHIT11. When the amount of SeCBD remained constant, the amount of chitinase activity increased with increases in SeCHIT7N and SeCHIT11.

Further investigation on the effects of SeCBD on SeCHIT7N and SeCHIT11 indicated that the chitinase activity of SeCHIT7N and SeCHIT11 increased significantly when the proportion of SeCBD increased, although after the ratio of SeCHIT7N and SeCHIT11 to SeCBD reached 1:10 and 1:20, the chitinase activity did not increase significantly (Figure 4D).

Taken together, the above results illustrate that infection with HvAV-3h not only seriously influences spatiotemporal specific transcription and expression of *SeCHIT*s but also disturbs the transcription and expression of *SeCBD*, influencing the chitinase activity in larvae.

## Discussion

Insect chitinases play an indispensable role in insect growth and development. Once the transcriptional pattern or activity of chitinase was interfered with, the effect is irreversible in the host insect. It was observed by Zhu et al. (2008a) that *T. castaneum* pupae were unable to complete adult eclosion after the transcript level of *T. castaneum* chitinase 5 (*TcCHIT5*) was down-regulated following injection of *TcCHIT5*-specific dsRNA into the larvae. After specific knockdown of *TcCHIT10* in *T. castaneum*, the eggs successfully hatch, and larvae are able to molt and pupate, but metamorphosis into the adult stage is blocked. Abnormal contractions and wing/elytra abnormalities were also noted in *TcCHIT7*-silenced larvae and pupae (Zhu et al., 2008a). In *Chilo suppressalis*, knockdown by chitinase 1 (*CsCHIT1*) resulted in adults with curled wings, whereas silencing of *C. suppressalis* chitinase 2 (*CsCHIT2*) or *C. suppressalis* chitinase 4 (*CsCHIT4*) arrested molting. Defects in pupation occurred when transcription of *C. suppressalis* chitinase 3 (*CsCHIT3*) interfered with dsRNA (Su et al., 2016). After interference in the transcription of *Panonychus citri* chitinase 1 (*PcCHIT1*) in the citrus red mite, *Panonychus citri* (McGregor) (Acari: Tetranychidae), the treated larvae and nymphal stages had a very depressed molting rate compared to the controls (Xia et al., 2016). These results suggest that insect (and mite) growth and development is likely to be seriously affected when the normal transcription of insect chitinase is disturbed by external factors. Our results showed that the slow growth observed in larvae infected by HvAV-3h is likely a consequence of the original transcriptional pattern of *SeCHIT7* and *SeCHIT11* being seriously disrupted by the virus.

The majority of insect pathogens are able to encode chitinase in order to infect the host more efficiently, self-replicate, or become more widespread in the infection process. Examples include Bacteria (Chernin et al., 1997; Sang et al., 1997; Tsujibo et al., 1998), fungi (De la Cruz et al., 1992; Felse and Panda, 2000; Omumasaba et al., 2001; Wang et al., 2002), actinomycetes (Reynolds, 1954), and baculovirus (Ohkawa et al., 1994; Hawtin et al., 1995), all of which have genes encoding chitinases that facilitate the overwhelming of the insect’s defense mechanisms (PM or cuticle) during infection. Researchers often concentrate on the changes occurring in chitinase encoded by a pathogen but ignore changes of chitinase encoding by the host insect during this process. The presence of chitinases encoded by pathogens can make studies involving the effects of pathogens on host-encoded chitinases difficult. The end result is that many of the studies involving a pathogen’s influence on host chitinases are incomplete. Because HvAV-3h did not encode chitinase, we were provided with an excellent system to study the effects of pathogens on chitinases encoded by a host. By comparing the chitinase activity in whole-body samples of mock-infected larvae with those infected with HvAV-3h, we determined that HvAV-3h significantly inhibited chitinase activity at 48–168 hpi. Chitinase activity was also inhibited in the four tissues we tested separately at 144–168 hpi, especially in the fat body and cuticle. The transcription and expression levels of *SeCHIT7*, *SeCHIT11*, and *SeCBD* in mock-infected and HvAV-3h-infected larvae were also investigated, showing that although HvAV-3h increased the transcriptional level of *SeCHIT7* and *SeCHIT11*, it inhibited the transcription and expression of *SeCBD*, preventing an increase in chitinase activity at 120–168 hpi. Previous studies have shown that the fat body and cuticle are the tissues that are most readily affected by HvAV-3h (Carner and Hudson, 1983; Hamm et al., 1998). Our research indicated that the transcription of *SeCHIT7* was markedly inhibited at 144 and 168 hpi in the fat body and cuticle and that *SeCHIT11* was dramatically inhibited at 168 hpi in the cuticle. While the expression of SeCBD was mainly detected in healthy larval fat body and cuticle, it could not be detected at all in HvAV-3h-infected larvae. These results show that HvAV-3h significantly inhibited chitinase activity during this period. After HvAV-3h infection, the virus had seriously damaged the fat body and cuticle at 144–168 hpi, which is the key time period for the host to complete its pupation. Apparently, infection by HvAV-3h results in host larvae being unable to complete their metamorphosis from larvae to pupae, although further research will be necessary to confirm this speculation.

The typical characteristic of larvae infected by baculovirus, an important insect virus, is liquefaction in their later stages. This condition is closely related to that observed after chitinase disruption. The first insect baculovirus chitinase gene was reported from the genome of Autographa californica nuclear polyhedrosis virus (AcMNPV) (Ayres et al., 1994). Current research has shown that chitinases encoded by viruses play an important role in their infection and proliferation. Examples of this include the following. I. Association with viral infection mechanisms. This may be caused by baculovirus chitinase being secreted into the insect midgut and subsequently destroying the constitutive chitin component of the midgut PM. This would likely result in the midgut epithelium to the intestine being exposed. Previous research showed that AcMNPV chitinase could decompose the PM in the alkaline environment of the larval midgut, enabling the polyhedral virus particles to effectively contact the epithelial cells of the midgut (Hawtin et al., 1997). II. Hydrolyzing the chitin in the host to promote liquefaction of the larvae, thereby promoting further spread of the virus. The chitinase of AcMNPV played a direct role in the process of liquefying the host tissues in the late stage of infection of *Trichoplusia ni*. This condition would ultimately degrade the epidermis of the larvae, enabling the polyhedra to be released from the host to spread the disease (Hawtin et al., 1997). The same baculovirus chitinase of Bombyx mori nucleopolyhedrovirus (BmNPV) transcribed by BmNPV is the main cause of tissue liquefaction in silkworms (Daimon et al., 2007).

HvAV-3h does not encode chitinases. On the one hand, the virus has a low degree of efficiency when introduced orally, which helps to explain why transmission of the virus is dependent on injection by parasitoid wasps via oviposition (Li et al., 2015). On the other hand, HvAV-3h infection cannot cause liquefaction of the host larvae. This indicates that the presence of the virus leads to a significant decline in chitinase activity in host larvae. When the chitinase activity continues at a lower level, the infected larvae were not able to complete the transformation from larvae to pupae. When the larvae state is maintained for a prolonged period of time, the probability of parasitism by parasitic wasps would be increased. This conjecture, however, will require further study. HvAV-3h does not completely inhibit the chitinase activity of larvae, but it is controlled within a certain range. This statement is consistent with our observations: the mock-infected larvae can develop normally and enter the pupal stage, and although the infected larvae are able to molt, they are unable to initiate pupation and eventually die. In addition, in infected larvae, the durations of the later instars were longer than in mock-infected larvae.

In summary, detection of transcriptional profiles of *SeCHIT*s and chitinase activity in both mock-infected and HvAV-3h-infected *S. exigua* larvae indicated that infection by HvAV-3h affects the transcriptional pattern and chitinase activity in *S. exigua* larvae. This causes the inability of the host larvae to complete their development while remaining in the larval state for extended periods of time. Our research will provide important information with which to better understand the influence of HvAV-3h on chitinase activity during host infection. This work is only the beginning, and many in-depth follow up studies should be performed, such as investigations centered on how *SeCHIT*s are regulated in HvAV-3h infections and determining the role of chitinase in the infection and diffusion of HvAV-3h.

## Data Availability Statement

The datasets generated for this study are available on request to the corresponding author.

## Author Contributions

G-HH contributed to the study design. LH, Y-YO-Y, NL, YC, and S-QL performed the experiments. LH and G-HH contributed reagents. LH and Y-YO-Y analyzed the data. LH, Y-YO-Y, NL, YC, S-QL, and G-HH wrote the manuscript. All authors read and approved the final manuscript.

## Conflict of Interest

The authors declare that the research was conducted in the absence of any commercial or financial relationships that could be construed as a potential conflict of interest.

## Footnotes

1 https://www.ncbi.nlm.nih.gov/orffinder/
